# Diagnostic and prognostic utility of tissue factor for severe sepsis and sepsis-induced acute lung injury

**DOI:** 10.1186/s12967-015-0518-9

**Published:** 2015-05-30

**Authors:** Mingming Xue, Zhan Sun, Mian Shao, Jun Yin, Zhi Deng, Jin Zhang, Lingyu Xing, Xiaoliang Yang, Bin Chen, Zhimin Dong, Yi Han, Si Sun, Yuxin Wang, Chenling Yao, Xun Chu, Chaoyang Tong, Zhenju Song

**Affiliations:** Department of Emergency Medicine, Zhongshan Hospital, Fudan University, 180 Fenglin Road, Shanghai, 200032 PR China; Department of Genetics, Chinese National Human Genome Center, 250 BiBo Road, Shanghai, 201203 PR China

**Keywords:** Severe sepsis, Acute respiratory distress syndrome, Tissue factor, Tissue factor pathway inhibitor

## Abstract

**Background:**

Tissue factor (TF) and tissue factor pathway inhibitor (TFPI) play a central role in the endothelial permeability regulation and dysfunction, which is associated with the development of sepsis and acute lung injury/acute respiratory distress syndrome (ALI/ARDS). The aim of this study is to assess the diagnostic and prognostic values of TF and TFPI in patients with sepsis and sepsis-induced ARDS.

**Methods:**

A total of 62 patients with sepsis, 167 patients with severe sepsis and 32 healthy volunteers were enrolled in this prospective observational study. TF and TFPI levels were measured by enzyme-linked immunosorbent assay (ELISA).

**Results:**

Patients with sepsis-induced ARDS showed significantly higher median levels of TF compared with patients without ARDS (1425.5 (1019.9 to 2595.2) pg/ml *vs* 916.2 (724.1 to 1618.2) pg/ml, *P* < 0.001), and compared with sepsis patients (943.5 (786.4 to 992.4) pg/ml, *P* < 0.001) on the day of admission. However, there was no significant difference between sepsis patients and healthy subjects, or between septic shock and non-septic shock patients (*P* > 0.05). The AUC of TF for the diagnosis of sepsis-induced ARDS was 0.749 (95% confidence interval (CI) 0.675-0.822). Plasma TF levels in the non-survivors of severe sepsis were significantly higher than those of survivors (1618.6 (1017.1 to 2900.8) pg/ml *vs*. 979.9 (757.2 to 1645.5) pg/ml, *P* < 0.001), and multivariate logistic regression showed the plasma value of TF was the independent predictor for 30-day mortality in patients with severe sepsis (*P* = 0.0022, odds ratio (OR) = 1.41, 95% CI 1.24-1.69). The AUC of TF for predicting 30-day mortality in severe sepsis patients was 0.718 (95% CI 0.641-0.794). However, there was no significant difference in the plasma TFPI values among the healthy control, sepsis and severe sepsis groups (*P* > 0.05).

**Conclusions:**

Our data showed that tissue factor is a valuable diagnostic biomarker for the diagnosis of sepsis-induced ARDS. Moreover, tissue factor is a strong prognostic marker for short-term mortality in severe sepsis and sepsis-induced ARDS patients.

## Introduction

Despite advances in the development of numerous drugs and supportive care therapies, severe sepsis remains an unconquered challenge for clinical investigators and physicians with an unacceptable high mortality rate of 28% to 50%. Sepsis is the most common cause of death in the non-cardiac intensive care unit (ICU) [1]. The pathogenesis of sepsis is not precisely understood; emerging evidence suggested that an exaggerated systemic host inflammation and coagulation response to infectious pathogens led to microvascular thrombosis and multiple organ dysfunction syndromes (MODS). In recent years, mounting empirical evidence supported that an extensive cross-talk between the inflammation and coagulation systems played a pivotal role in the pathogenesis of microvascular failure and subsequent multiple organ failure, as a result of severe infection [2]. Pro-inflammatory cytokines lead to activation of coagulation and downregulate the physiologic anticoagulant pathways; conversely, activated coagulation proteases modulate the inflammatory response [3, 4]. Many clinical studies found that virtually all septic patients had coagulation abnormalities. The clinical manifestations of coagulation abnormalities were highly variable, depending on the illness severity and duration time of infection. Most sepsis patients only had clinically insignificant changes in platelet count. However, uncontrollable clot formation and bleeding in overt disseminated intravascular coagulation (DIC) was seen in a few severe sepsis and septic shock patients. It is clearly established that the overexpression of TF or the imbalance between TF and TFPI was closely related with the mechanisms involved in the pathological derangement of coagulation in septic patients [5–7].

TF, a transmembrane glycoprotein, is expressed by various cell types [8]. In endotoxemic and septic animals, TF expression is increased not only in monocytes-macrophages but also in tissue cells, e.g. lung and kidney epithelial cells, and brain astrocytes [9, 10]. Many studies have suggested that the aberrant in vivo expression of TF plays a pivotal role in sepsis-associated blood clotting change, as indicated by the following observations: 1) the impairment of the TF pathway by various means prevents coagulation abnormalities and lethality in animal models of sepsis or endotoxemia [9–11]; 2) the plasma levels of TF are increased in septic patients and generally associated with raised concentrations of markers of clotting activation [9, 11, 12]. Normally, thrombin generation via the TF pathway is rapidly controlled by TFPI, which is an endogenous inhibitor of TF-associated coagulation cascade [13]. TFPI directly inhibits activated factor X and, in a factor-dependent manner, produces feedback inhibition of the factor VIIa/TF complex. It has been reported that the lower levels of TFPI are strongly correlated with organ dysfunction as well as worse outcome of severe sepsis [14].

Considering the potential association of an activated coagulation system with sepsis pathophysiology, particularly the role of TF pathway as an important initiator of the coagulation system, TF and TFPI are excellent candidate biomarkers for early diagnosis of sepsis, risk stratification, and evaluation of prognosis in septic patients. Although some clinical studies found the plasma TF and TFPI levels are significantly changed in septic patients and correlated with the severity of sepsis, the results were controversial because of small sample size. Thus, the objective of this study was to assess the prognostic and diagnostic value of plasma TF and TFPI levels in patients with sepsis, sepsis-induced ARDS and septic shock in a relatively large prospective study.

## Materials and methods

### Study population

From March 2010 to December 2013, a total of 62 patients with sepsis, 167 patients with severe sepsis admitted into the Emergency department and ICU of Zhongshan Hospital, Fudan University (Shanghai, China) were included. Definitions of sepsis, severe sepsis and ARDS were in accordance with the American College of Chest Physicians/Society of Critical Care Medicine Consensus Conference, the American-European Consensus Conference statements and the new (Berlin) definition [15–17]. Severe sepsis subjects enrolled had either organ dysfunction or septic shock. Exclusion criteria included age < 18 years, pregnancy, diffuse alveolar hemorrhage, severe chronic respiratory disease, directive to withhold intubation, severe chronic liver disease (defined as a Child-Pugh score of > 10), malignancy, using of chronic high-dose immunosuppressive therapy (steroids with equivalent prednisone ≥ 0.5 mg/kg per day or cytotoxic agents for immunologic disorders) and Acquired Immune Deficiency Syndrome (AIDS) patients. Clinical and demographic data at baseline, including Acute Physiology and Chronic Health Evaluation (APACHE) II scores, organ failure, previous health status, hospital and ICU mortality were obtained after the patient met inclusion criteria. This study was approved by the Ethic Committee of Zhongshan Hospital, Fudan University, Shanghai, China (Record no: 2006–23). Informed consent was obtained from subjects or from their legal surrogates before enrollment.

### Data collection and the TF/TFPI measure

Demographic and clinical data were recorded on the study enrollment and included age, gender, PaO_2_/FiO_2_ and APACHE II score. In addition, the degree of hypoxemia of ARDS was divided into mild (200 < PO_2_/FiO_2_ ≤ 300), moderate (100 < PO_2_/FiO_2_ ≤ 200) and severe (PO_2_/FiO_2_ ≤ 100) according to the Berlin definition. Blood samples for determination of TF/TFPI were collected at enrollment for all participants and were centrifuged within the next 1 hour. Plasma samples were frozen at −80 °C for further analysis. Levels of TF/TFPI were measured using an ELISA kit according to the manufacturer’s instructions (Human TF/TFPI ELISA Kit, R&D, Minnesota, USA). The plates were read at a wavelength of 450 nm with an automatic ELISA reader and the assay did not cross-react with other related protein.

### Statistical analysis

Continuous variables were presented as median (interquartile range), and categorical variables as numbers and percentages. For multi-group comparisons, Kruskal-Wallis one-way analysis of variance was applied, and two-group comparisons were performed nonparametrically using the Mann–Whitney U test. To compare the predictive value of TF, TFPI and APACHE II score for sepsis, severe sepsis, septic shock, sepsis-induced ARDS and 30-day mortality, receiver operating characteristic (ROC) curves were constructed and the areas under the ROC curves (AUCs) were determined. On the basis of optimal thresholds determined according to ROC curve analysis, prognostic parameters (sensitivity, specificity) were also calculated. The optimal cutoff value was determined when the Youden index reached the maximum value. Logistic regression was assessed by univariate and multivariate analysis to identify independent predictors of outcome. The survival estimate was based on the Kaplan-Meier product-limit method, and comparisons of survival distributions were based on the log-rank test. All probabilities were two tailed and *P* < 0.05 was regarded as significant. Data were statistically analyzed with SPSS 17.0 software (SPSS Inc., Chicago, IL, USA).

## Results

### Study population characteristics

A total of 62 sepsis and 167 severe sepsis patients in the Emergency department and ICU of Zhongshan Hospital and 32 healthy controls were enrolled in this study. Severe sepsis patients were classified into ARDS and non-ARDS groups, septic shock and non-septic shock groups, survivor and non-survivor groups according to the illness severity and 30-day survival. The baseline characteristics of the study population were presented in Table 1. No significant difference was found in age and gender among the three groups (healthy control, sepsis and severe sepsis groups). The APACHE II scores in the severe sepsis, sepsis-induced ARDS, septic shock, non-survivor patients were significantly higher than those in patients with sepsis, non-ARDS, non-septic shock and survivor respectively (*P* < 0.05). Sixty-five percent of severe sepsis patients had positive microbial isolates: 39% of the positive isolates were gram-positive, 52% gram-negative, and 9% fungal. In patients with positive isolates, the most common gram-positive organisms were Staphylococcus aureus and Staphylococcus epidermidis; the most common gram-negative organisms were Acinetobacter baumannii and Escherichia coli.

**Table 1:** The baseline characteristics of the study populations

	Number	Age (Years)	Male (N,%)	APACHE II score	P value (APACHE II score)
Healthy control	32	62 (56–66)	18 (56.3%)	3 (3–5)	
Sepsis patients	62	65 (56–75.25)	35 (56.5%)	10 (9–12)	P < 0.001(severe sepsis *vs* sepsis)
Severe sepsis patients	167	65 (55–74)	101 (60.5%)	15 (12–20)	
Sepsis-induced ARDS	73	64 (53.5-72)	45 (61.6%)	16 (13–21)	P = 0.002 (ARDS *vs* non-ARDS)
Non-ARDS	94	65 (56–76)	56 (59.6%)	14 (12–16.5)	
Septic shock	54	66 (56–76)	33 (61.1%)	17.5 (14–21)	P = 0.003 (shock *vs* non-shock)
Non-septic shock	113	65 (55–71)	68 (60.2%)	14 (12–17)	
Survivor	98	65 (56–74)	93 (60.2%)	12 (14–16)	P < 0.001 (survivor *vs* non-survivor)
Non-survivor	69	65 (47–73)	42 (60.9%)	18 (14–21)	
Gram positive	42	63 (54.5-73)	24 (57.1%)	15 (12–20)	P = 0.94 (G^+^ *vs* G^−^)
Gram negative	56	65 (54.25-74.75)	30 (60.7%)	15 (12–19)	

### The plasma TF values in sepsis-induced ARDS patients

**Fig. 1:**
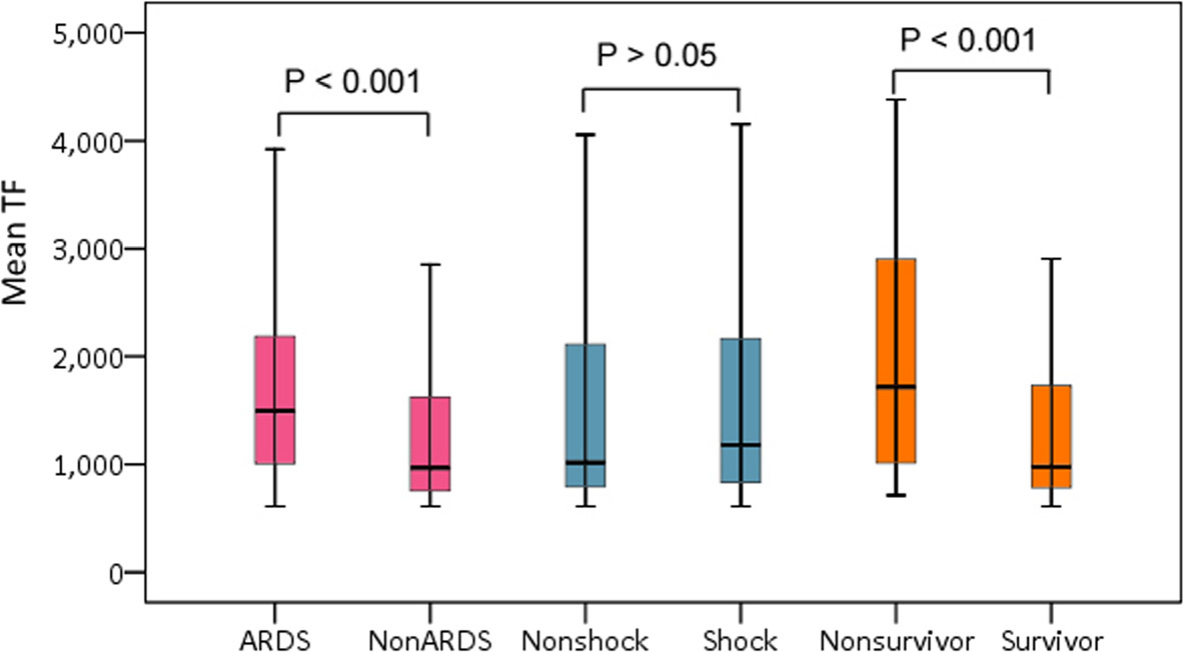
The plasma values of TF in different subgroup patients with severe sepsis. Patients with sepsis-induced ARDS had significantly higher median levels of TF compared with patients without ARDS at the time of diagnosis (*P* < 0.001), however there was no significant difference between septic shock and non-septic shock groups. The TF plasma values in the non-survivors of severe sepsis were significant higher than those in the survivors (*P* < 0.001)

**Fig. 2:**
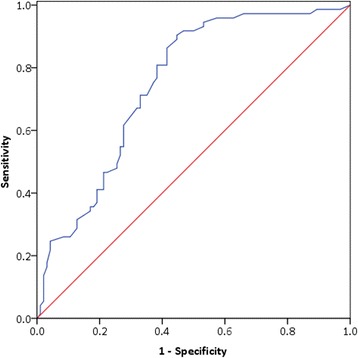
The ROC curve for TF in relation to the diagnosis of sepsis-induced ARDS. The AUC for TF in relation to the diagnosis of sepsis-induced ARDS from non-ARDS patients was 0.749 (95% CI 0.675-0.822)

Table 2 showed the plasma levels of TF and TFPI in the healthy subjects, sepsis and severe sepsis patients. There were no statistical differences in the TFPI levels among the healthy control, sepsis and severe sepsis groups (*P* > 0.05). The plasma TF levels of patients with severe sepsis were markedly higher than those in both healthy controls and sepsis patients (*P* < 0.001), whereas there was no significant difference between the healthy subjects and sepsis patients (*P* > 0.05). In the sub-group analysis, patients with sepsis-induced ARDS had significantly higher median levels of TF compared with patients without ARDS (1424.5 (1019.9 to 2595.2) pg/ml *vs*. 916.2 (724.1 to 1618.2) pg/ml, *P* < 0.001) and compared with sepsis patients (943.5 (786.4 to 992.4) pg/ml, *P* < 0.001) at the time of diagnosis, however there was no significant difference between septic shock and non-septic shock patients (*P* > 0.05) (Fig. 1). The AUC for TF in relation to the diagnosis of sepsis-induced ARDS from non-ARDS patients was 0.749 (95% CI 0.675-0.822) (Fig. 2). At a cutoff point > 1005.8 pg/ml, TF provided specificity of 80.8% and sensitivity of 61.7% for the diagnosis of sepsis-induced ARDS.

**Table 2:** The plasma values of TF and TFPI in healthy, sepsis and severe sepsis groups

	Number	TF (pg/ml)	TFPI (pg/ml)	P value (TF)
Healthy control	32	870.1 (779.2-1049.7)	248612.6 (193776.8-339925.5)	P = 0.33 (healthy control *vs* sepsis)
Sepsis	62	943.5 (786.4-992.4)	300279.5 (181779.2-349692.7)	
Severe sepsis	167	1045.7 (834.2-2156.6)	314059.7 (203476.4-317680.2)	P = 2.11 × 10^−4^ (severe sepsis *vs s*epsis)
Sepsis-induced ARDS	73	1424.5 (1019.9-2595.2)	317477.4 (195388.2-317783.1)	P = 3.66 × 10^−8^ (ARDS *vs* Non-ARDS)
Non-ARDS	94	916.2 (724.1-1618.2)	311405.5 (179876.1-352123.9)	
Septic shock	54	1182.3 (834.2-2164.8)	316321.6 (190792.5-350043.2)	P = 0.541 (septic shock *vs* non-shock)
Non-shock	113	1022.7 (798.6-2166.5)	309326.5 (171236.8-304562.7)	
Survivor	98	979.9 (757.2-1645.5)	336934.1 (198865.2-343567.5)	P = 9.25 × 10^−6^ (survivor *vs* non-survivor)
Non-survivor	69	1618.6 (1017.1-2900.8)	335995.4 (181790.1-340065.2)	
Gram positive	42	1113.7 (831.1-2352.8)	318765.9 (203712.4-321100.6)	P = 0.419 (G^+^ *vs* G^−^)
Gram negative	56	1521.6 (911.2-2464)	320135.5 (190006.3-356432.5)

**Fig. 3:**
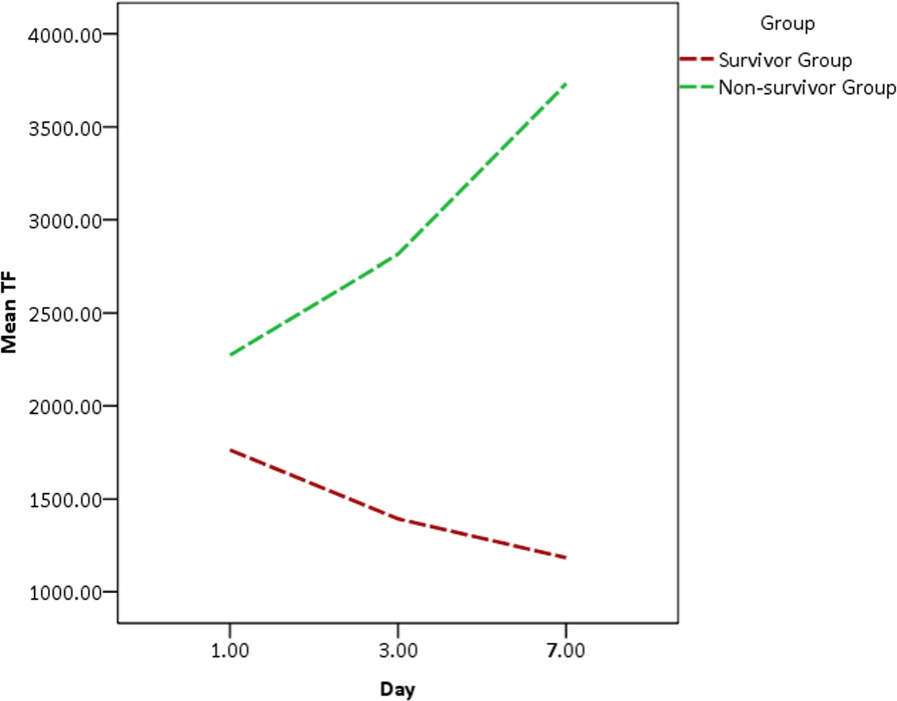
The plasma levels of TF on the 1st, 3rd, 7th day of sepsis-induced ARDS patients. On the day of admission, the TF plasma levels in non-survivors of ARDS patients were significantly elevated compared with those in survivors of ARDS patients (*P* = 0.00013). The TF levels in ARDS survivor were declined obviously on the 3rd and 7th day; however, the TF values in the non-survivors were increased continually on the 3rd and 7th day

Then, the plasma TF levels in 60 sepsis-induced ARDS patients were measured on the 1st, 3rd and 7th day after ARDS diagnosis. On the day of admission, the TF plasma levels in non-survivors of ARDS patients were significantly elevated compared with those in survivors of ARDS patients (2163.3 pg/ml *vs*. 1666.6 pg/ml, *P* = 0.00013). The TF levels in ARDS survivor were declined obviously on the 3rd and 7th day; however, the TF values in the non-survivors were increased continually on the 3rd and 7th day (Fig. 3). Then, all sepsis-induced ARDS patients were classified into mild (n = 11), moderate (n = 21) and severe (n = 41) groups according to the degree of hypoxemia. There were significant differences in TF value among the mild, moderate and severe ARDS groups (Table 3). The TF values of the severe and moderate groups were significantly higher than those of the mild group (*P* < 0.001) (Table 3). All these results indicated that the plasma levels of TF were obviously associated with the illness severity and outcome in sepsis-induced ARDS patients.

**Table 3:** The TF plasma values in the mild, moderate and severe sepsis-induced ARDS patients

	Number	TF (pg/ml)	P value
Mild ARDS	11	967.7(897.0-1008.7)	P = 0.0002(mild *vs* moderate)
Moderate ARDS	21	1234.0(1076.9-1666.6)	P = 0.001(moderate *vs* severe)
Severe ARDS	41	2170.0(1234.3-3340.0)	P = 2.25 × 10-6 (mild *vs* severe)

### The association between the plasma TF levels and outcome of severe sepsis

**Fig. 4:**
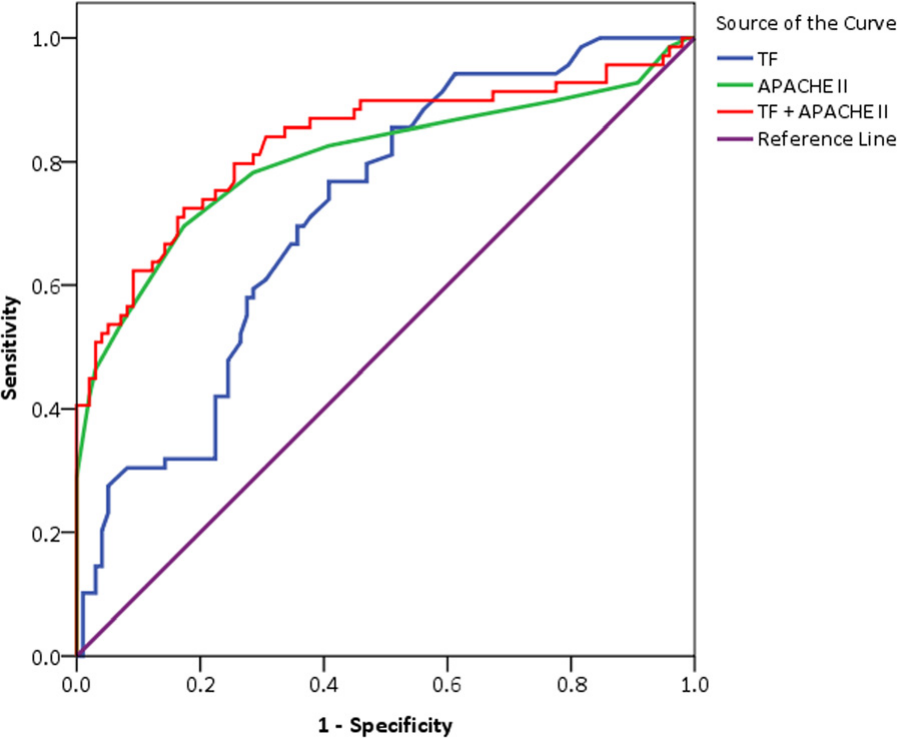
The ROC curve for TF, APACHE II in relation to the outcome of severe sepsis patients. The AUC of TF for predicting 30-day mortality in septic patients was 0.718, slightly lower than that of APACHE II scores 0.804. The AUC of TF in combination with the APACHE II scores was 0.832, which was more statistically significant compared with TF alone

**Fig. 5:**
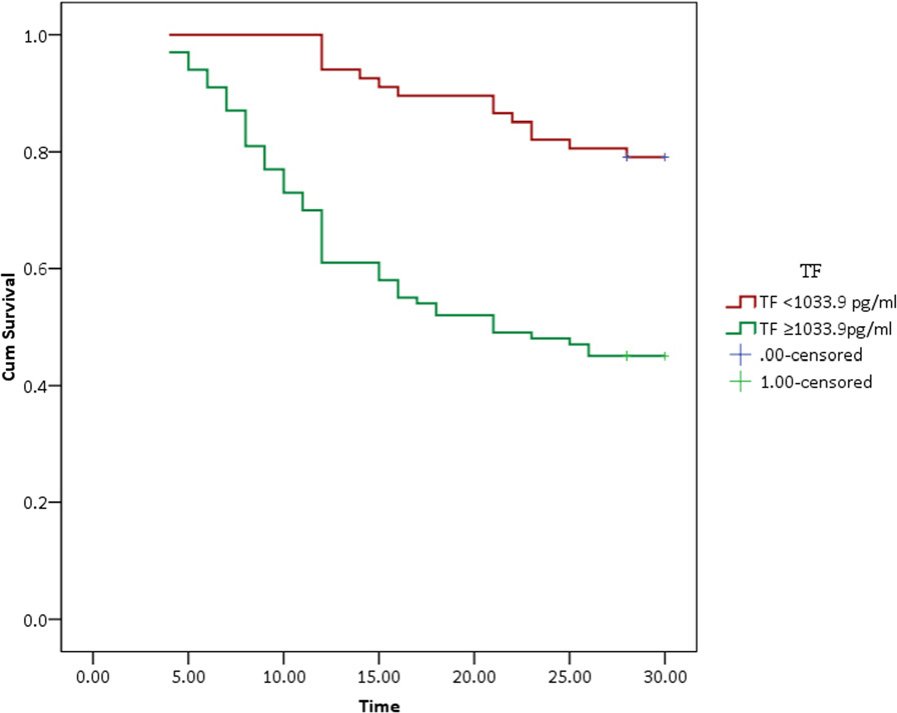
The survival probability of severe sepsis patients by TF value. A Kaplan-Meier curve was drawn according to the value of 1033.9 pg/ml for TF as a cutoff point to describe death over 30 days of follow-up

Moreover, our results also showed that the TF plasma values were associated with the outcome of severe sepsis. The TF plasma values in the non-survivors of severe sepsis were significant higher than those in the survivors (1618.6 (1017.1- 2900.8) pg/ml *vs*. 979.9 (757.2-1645.5) pg/ml, *P* < 0.001) (Table 1, Fig. 1). Univariate analysis showed that admission plasma TF levels and the APACHE II scores were the both predictors of 30-day mortality in patients with severe sepsis. Multivariate logistic regression analysis showed the TF levels remained the independent predictor for mortality after adjustment for APACHE II scores (*P* = 0.0022, OR = 1.41, 95% CI 1.24-1.69). The ROC was drawn to evaluate the value of TF to predict 30-day mortality. The AUC of TF for predicting 30-day mortality in septic patients was 0.718 (95% CI 0.641-0.794), slightly lower than that of the APACHE II scores (0.804 (95% CI 0.730-0.878), *P* < 0.05). The AUC of TF in combination with the APACHE II scores was 0.832 (95% CI 0.764-0.900), which was more statistically significant compared with TF alone (Fig. 4). The optimal cut off value for predicting death was > 1033.9 pg/ml, which gave specificity of 62.5% and sensitivity of 71.1%. A Kaplan-Meier curve was drawn according to the value of 1033.9 pg/ml for TF as a cutoff point to describe death over 30 days of follow-up (Fig. 5).

## Discussion

Several clinical studies found the plasma TF levels were significantly changed in septic and ARDS patients; however, the sample sizes were relatively small [18–21]. Our current study with a relatively large-size sample demonstrated that patients with sepsis-induced ARDS had markedly high tissue factor levels compared with patients without ARDS. And, the plasma levels of TF were significantly associated with the illness severity and outcome in sepsis-induced ARDS patients. Moreover, our results also indicated that the plasma values of TF in the non-survivors with severe sepsis were obviously higher than those in the survivors. Univariate analysis showed both plasma TF levels and APACHE II score were the predictors for 30-day mortality in patients with severe sepsis and further logistic regression analysis revealed the TF levels were independent of APACHE II score. APACHE II score in the septic shock was significantly higher than those in patients with non-septic shock. However, there was no significant difference between septic shock and non-septic shock patients in the plasma tissue factor levels.

Several studies proved that the plasma concentration of TF was significantly higher in both ARDS and severe sepsis patients [18, 19]. One prospective cohort study consisting of 113 patients (27 patients with ARDS, 31 patients at risk but not developing this syndrome, and 55 patients without ARDS) found that the values of TF in patients with ARDS were significantly elevated compared with those measured in other groups [20]. The TF levels were significantly correlated with lung injury score. In addition, TF dependent coagulation pathway of plasma was extensively activated in patients with ARDS. Further supporting data demonstrated that the procoagulant properties of broncoalveolar lavage fluid (BALF) from ARDS patients were the result of TF induction, and further indicated that BALF neutrophils were a main source of TF in intra-alveolar fluid [21]. All these results were in accord with the current study that showed the plasma levels of TF were associated with the illness severity and outcome in sepsis-induced ARDS patients. TF was a valuable diagnostic biomarker for the diagnosis of sepsis-induced ARDS. In addition, TF was a strong prognostic marker for short-term mortality in severe sepsis and sepsis-induced ARDS patients.

Activation of coagulation through the TF-dependent pathway has been suggested to be a central mechanism in the pathogenesis of ALI and other inflammatory conditions including pneumonia, sepsis, and ventilator-induced lung injury [22]. Extravascular fibrin deposition is a common characteristic of the acute inflammatory response and is prominent in the alveolar compartment of patients with ARDS. Fibrin deposition in the lung is regulated by a balance of locally expressed pathways of coagulation and fibrinolysis [23]. Although activation of the contact and intrinsic pathway occurs, fibrin deposition within the injured alveolar compartment is mainly initiated by locally increased activity of the extrinsic coagulation pathway. Tissue factor is the initiator of the extrinsic coagulation pathway. Previous studies found that in the early stage of acute lung injury and inflammation, TF protein levels were significantly increased in the alveolar spaces of the lung [24]. During sepsis and ARDS, a variety of stimuli such as proinflammatory cytokines and shear stress induce TF expression on monocytes and endothelial cells. When TF expresses on the cell surface, factor VIIa binds to TF, forming the bianry of TF/FVIIa. Then, the bianry of TF/FVIIa binds to factor X, forming the ternary TF/FVIIa /FXa complexes, and leading the eventual thrombin generation and fibrin formation. It is well established that TF has widespread implications in the human body beyond its function in the coagulation system. As an intermediate for factor VIIa, TF also played an important role in the intracellular signaling of established inflammatory pathways. These effects were mediated by protease-activated receptors (PARs), members of the G protein-coupled receptor family that activate mitogenactivated protein kinase and NF-κB signaling. Until now, a total of four subtypes of PARs have been identified. PARs form the molecular link between coagulation and inflammation. Among them, the PAR1 and PAR2 were implicated in the acute inflammatory response. [25]. Pawlinski and Mackman [7] proved that genetically modified mice expressing low levels of TF exhibited reduced interleukin-6 expression and increased survival in a mouse model of endotoxemia compared with control mice.

Some animal experiments and clinical studies proved the benefit of the early blockade of the TF-VIIa-activated coagulation system in reducing both systemic and pulmonary inflammation as well as coagulation, and in improving lung physiology, histological results and even survival. He *et al.* found that anti-human TF antibody attenuated the severity of lung tissue injury, reduced alveolar fibrin deposition and protein concentration in BALF using ARDS model induced by intestinal ischemia-reperfusion [26, 27]. In addition, Welty-Wolf and colleagues found that systemic blockade of factor X binding to the tissue factor-factor VIIa complex could attenuate fibrinogen depletion, decrease proinflammatory cytokines and prevent sepsis-induced damage to the lungs [28].

Normally, thrombin generation via the TF pathway is rapidly controlled by TFPI [13]. TFPI is the main regulator in the initial step of the coagulation cascade mediated by TF, which binds to coagulation factors Xa, VIIa, TF and forms an inactive complex. It has been reported that the imbalance between the levels of TF and TFPI seems to play a pivotal role in the pathogenesis of sepsis and ARDS. A prospective, cohort study recruited 31 consecutive patients with sepsis, classified as 15 survivors and 16 non-survivors, and 10 healthy volunteers served as controls. They found activation of TF-dependent coagulation pathway not adequately balanced by TFPI has important roles in sustaining DIC and systemic inflammatory response syndrome [29]. However, in our sample the plasma TFPI values among the healthy controls, sepsis and severe sepsis groups were not significantly different.

There were several limitations to our study. First, the ARDS patients enrolled in the current study were sepsis-induced patients. We did not measure the TF levels in other risk factors induced-ARDS patients. Second, the absence of a single-objective gold standard for diagnosis of ARDS was a challenge inherent to all studies of diagnostic testing for this syndrome regardless of the new Berlin definition used. Finally, we did not test the concentration of TF in alveolar fluid and lack an extended comparison with other biomarkers of ALI/ARDS.

## Conclusions

In this study, TF level in the patients with sepsis-induced ARDS was significantly higher than that of non-ARDS patients. And, it was associated with the clinical severity of ARDS according to the new Berlin definition. Thus, the present study provided further evidence about TF as a valuable diagnostic biomarker for the diagnosis of sepsis-induced ARDS. The plasma TF levels in combination with APACHE II scores were a strong prognostic predictor for patients with severe sepsis.

### Key messages

The present study delivered strong evidence about TF as a valuable diagnostic biomarker for the diagnosis of sepsis-induced ARDS.

APACHE II scores and plasma TF levels at enrollment were the common predictors of 30-day mortality in patients with severe sepsis.

## Abbreviations

AIDS: Acquired immune deficiency syndrome
ALI: Acute lung injury
APACHE: Acute physiology and chronic health evaluation
ARDS: Acute respiratory distress syndrome
AUC: Area under the ROC curve
CI: Confidence interval
DIC: Disseminated intravascular coagulation
ELISA: Enzyme linked immunosorbent assay
ICU: Intensive care unit
MODS: Multiple organ dysfunction syndromes
TF: Tissue factor
TFPI: Tissue factor pathway inhibitor
OR: Odds ratio
ROC: Receiver operating characteristic
BALF: Broncoalveolar lavage fluid

## References

[1] Angus DC, van der Poll T (2013). Severe sepsis and septic shock. N Engl J Med.

[2] Remick DG (2007). Pathophysiology of sepsis. Am J Pathol.

[3] Anas AA, Wiersinga WJ, de Vos AF, van der Poll T (2010). Recent insights into the pathogenesis of bacterial sepsis. Neth J Med.

[4] Levi M, Ten CH (1999). Disseminated intravascular coagulation. N Engl J Med.

[5] Levi M (2010). The coagulant response in sepsis and inflammation. Hamostaseologie.

[6] Levi M, Van Der Poll T (2013). Thrombomodulin in sepsis. Minerva Anestesiol.

[7] Pawlinski R, Mackman N (2004). Tissue factor, coagulation proteases, and protease-activated receptors in endotoxemia and sepsis. Crit Care Med.

[8] Edgington TS, Mackman N, Brand K, Ruf W (1991). The structural biology of expression and function of tissue factor. Thromb Haemost.

[9] Semeraro N, Ammollo CT, Semeraro F, Colucci M (2010). Sepsis-associated disseminated intravascular coagulation and thromboembolic disease. Mediterr J Hematol Infect Dis.

[10] Pawlinski R, Mackman N (2010). Cellular sources of tissue factor in endotoxemia and sepsis. Thromb Res.

[11] Levi M (2008). The coagulant response in sepsis. Clin Chest Med.

[12] Gando S, Nanzaki S, Sasaki S, Kemmotsu O (1998). Significant correlations between tissue factor and thrombin markers in trauma and septic patients with disseminated intravascular coagulation. Thromb Haemost.

[13] Broze GJ (2003). The rediscovery and isolation of TFPI. J Thromb Haemost.

[14] Tang H, Ivanciu L, Popescu N, Peer G, Hack E, Lupu C (2007). Sepsis-induced coagulation in the baboon lung is associated with decreased tissue factor pathway inhibitor. Am J Pathol.

[15] American College of Chest Physicians/Society of Critical Care Medicine Consensus Conference: definitions for sepsis and organ failure and guidelines for the use of innovative therapies in sepsis. *Crit Care Med* 1992, 20:864–874. 1597042

[16] Bernard GR, Artigas A, Brigham KL, Carlet J, Falke K, Hudson L (1994). Report of the American-European consensus conference on ARDS: definitions, mechanisms, relevant outcomes and clinical trial coordination. The Consensus Committee. Intensive Care Med.

[17] Ranieri VM, Rubenfeld GD, Thompson BT, Ferguson ND, Caldwell E, Fan E (2012). Acute respiratory distress syndrome: the Berlin definition. JAMA.

[18] Liang Y, Li X, Zhang X, Li Z, Wang L, Sun Y (2014). Elevated levels of plasma TNF-alpha are associated with microvascular endothelial dysfunction in patients with sepsis through activating the NF-kappaB and p38 mitogen-activated protein kinase in endothelial cells. Shock.

[19] Gando S, Kameue T, Matsuda N, Hayakawa M, Morimoto Y, Ishitani T (2003). Imbalances between the levels of tissue factor and tissue factor pathway inhibitor in ARDS patients. Thromb Res.

[20] Gando S, Nanzaki S, Morimoto Y, Kobayashi S, Kemmotsu O (1999). Systemic activation of tissue-factor dependent coagulation pathway in evolving acute respiratory distress syndrome in patients with trauma and sepsis. J Trauma.

[21] Kambas K, Markiewski MM, Pneumatikos IA, Rafail SS, Theodorou V, Konstantonis D (2008). C5a and TNF-alpha up-regulate the expression of tissue factor in intra-alveolar neutrophils of patients with the acute respiratory distress syndrome. J Immunol.

[22] Welty-Wolf KE, Carraway MS, Ortel TL, Piantadosi CA (2002). Coagulation and inflammation in acute lung injury. Thromb Haemost.

[23] Bastarache JA, Wang L, Geiser T, Wang Z, Albertine KH, Matthay MA (2007). The alveolar epithelium can initiate the extrinsic coagulation cascade through expression of tissue factor. Thorax.

[24] Glas GJ, Van Der Sluijs KF, Schultz MJ, Hofstra JJ, Van Der Poll T, Levi M (2013). Bronchoalveolar hemostasis in lung injury and acute respiratory distress syndrome. J Thromb Haemost.

[25] Uusitalo-Jarvinen H, Kurokawa T, Mueller BM, Andrade-Gordon P, Friedlander M, Ruf W (2007). Role of protease activated receptor 1 and 2 signaling in hypoxia-induced angiogenesis. Arterioscler Thromb Vasc Biol.

[26] He X, Han B, Mura M, Li L, Cypel M, Soderman A (2008). Anti-human tissue factor antibody ameliorated intestinal ischemia reperfusion-induced acute lung injury in human tissue factor knock-in mice. PLoS One.

[27] He X, Han B, Bai X, Zhang Y, Cypel M, Mura M (2010). PTX3 as a potential biomarker of acute lung injury: supporting evidence from animal experimentation. Intensive Care Med.

[28] Welty-Wolf KE, Carraway MS, Ortel TL, Ghio AJ, Idell S, Egan J (2006). Blockade of tissue factor-factor X binding attenuates sepsis-induced respiratory and renal failure. Am J Physiol Lung Cell Mol Physiol.

[29] Gando S, Kameue T, Morimoto Y, Matsuda N, Hayakawa M, Kemmotsu O (2002). Tissue factor production not balanced by tissue factor pathway inhibitor in sepsis promotes poor prognosis. Crit Care Med.

